# Prevalence of medical factors related to aging among older car drivers: a multicenter, cross-sectional, descriptive study

**DOI:** 10.1186/s12877-022-03490-w

**Published:** 2022-10-11

**Authors:** Hideharu Hagiya, Ryosuke Takase, Hiroyuki Honda, Yasuhiro Nakano, Yuki Otsuka, Hitomi Kataoka, Mika Uno, Keigo Ueda, Misa Takahashi, Hiroko Ogawa, Yoshihisa Hanayama, Fumio Otsuka

**Affiliations:** Department of General Medicine, Dentistry and Pharmaceutical Sciences, Okayama University Graduate School of Medicine, 2-5-1 Shikata-cho, Kita-ku, Okayama, 700-8558 Japan

**Keywords:** Traffic safety, Older people, Aging, Motor vehicle accidents, Frailty, Dementia, Polypharmacy, Sarcopenia

## Abstract

**Aim:**

An increasing number of older adults in Japan are at an increased risk of road traffic crashes. This study aimed to investigate the prevalence of potential underlying medical factors that increase the risk of road traffic crashes among older people.

**Methods:**

This cross-sectional observational study was conducted in 11 medical institutions in Japan using self-administered questionnaires and physical examination from January to May 2021. The background and social data, data on the use of nursing care insurance, and clinical data suggestive of polypharmacy, sarcopenia, cognitive impairment, and frailty/oral frailty were obtained. The prevalence of these factors was compared between everyday and occasional drivers.

**Results:**

Data of 127 patients were collected; their median (interquartile range) age was 73 (70–78) years. Of the total participants, 82 were men (64.6%) and 45 were women (35.4%). There were 77 everyday drivers and 50 occasional drivers. Of these, 121 (95.3%) had not applied for nursing care insurance, but the numbers of those who required help 1 and 2 were 1 (0.8%) and 3 (2.4%), respectively. Prevalence of medical factors was as follows: polypharmacy, 27.6%; sarcopenia, 8.7%; dementia, 16.4%; frailty, 15.0%; and oral frailty, 54.3%; it was not significantly different between every day and occasional drivers. Intention to return the car license was significantly higher among the occasional drivers (2.6% vs. 14.0%; odds ratio: 6.7, 95% confidence interval: 1.2–70.6, *p* = 0.024).

**Conclusion:**

We uncovered the prevalence of medical factors that can be associated with road traffic crashes among Japanese older people aged ≥ 65 years in our community.

**Supplementary Information:**

The online version contains supplementary material available at 10.1186/s12877-022-03490-w.

## Introduction

Global aging is rapidly progressing, and the number of people aged 60 years is expected to reach 2 billion in 2050 [1]. Japan is one of the countries with a rapidly aging society at a pace unparalleled in the world. According to the data from the Ministry of Land, Infrastructure, Transport and Tourism, more than 19 million people aged ≥ 65 years held a driver’s license at the end of 2020 in Japan [2]. Mental and physical changes associated with aging are definitely linked with road traffic crashes among older drivers, which have recently become a social issue [3]. It is more pressing in the regional countryside where the need for car driving is higher than that in large cities with developed public transportation. Car driving requires advanced abilities, such as attentiveness, executive functions, and visuospatial cognitive ability, which decline with age, resulting in road traffic crashes.

Road traffic crashes frequently result in mortality in older drivers. A systematic review and meta-analysis suggested that older drivers have 3 to 20-fold higher risk of fatal crash than non-older drivers [4]. To reduce such road traffic crashes among older adults, since 2009, drivers in Japan aged ≥ 75 years have been screened for cognitive impairment when they renew their car license [5]. Then, the Road Traffic Law requires car drivers aged ≥ 70 years to attend a 2-h streamlined lecture when renewing their licenses. In 2017, the law made it mandatory for those aged > 75 years to take cognitive function tests when they intend to extend the licenses. Until then, authorities could only take measures to encourage their voluntary returns, and it was impossible to suspend the licenses of older car drivers unless there were extraordinary reasons. With this law revision, their driving license can be revoked or suspended if diagnosed with dementia. Furthermore, through the 2020 amendment, older adults aged > 75 years are required to take a driving skills test when renewing their license, in case they have committed certain violations in the past three years. Thus, various efforts have been made to prevent road traffic crashes among older drivers, including the revision of the law.

Various studies have revealed that medical factors are the underlying causes of road traffic crashes among older adults [4]. As people age, they experience multimorbidity and develop multiple diseases or conditions, which increase the risk of road traffic crashes [6]. Of the various factors, some may be preventable. For instance, the use of long-acting benzodiazepines was proven to be associated with an increased risk of road traffic crashes [7], which is avoidable. However, mental and physical dysfunctions associated with aging are difficult to ameliorate, and such factors should be appropriately evaluated in older drivers.

Hence, this study aimed to assess the prevalence of potential underlying medical factors that can be associated with road traffic crashes among older drivers. In particular, we compared these social and medical factors by the driving frequency.

## Methods

This was a cross-sectional observational study using self-administered questionnaires and a general physical examination performed between January and May 2021. Patients aged ≥ 65 years who visited the outpatient clinics of 11 medical institutions in Okayama, Kagawa, and Hiroshima prefectures in Japan (Okayama University Hospital, Marugame Medical Center, Kasaoka City Hospital, Kousei General Hospital, Okayama Memorial Hospital, Tamano City Hospital, Okayama Hakuaikai Hospital, Takahashi City National Health Insurance Nariwa Hospital, Niimi National Health Insurance Clinics [Yukawa Clinic], Mabi Memorial Hospital, and Okayama Kyokuto Hospital) were recruited.

### Definition

In addition to the baseline information such as age, gender, height, weight, and use of nursing care insurance, the following potential social factors that increase the risk of road traffic crashes were obtained: frequency of going out, the number of cohabiting family members, transportation most frequently used other than by car, walking time from home to the nearest station or bus stop, frequency of socializing with friends and peers, main source of daily news, daily concerns, visits to eye doctors and orthopedic surgeons, and use of hearing aids. The grade of the nursing care insurance in Japan is classified into requiring help (1 or 2) and requiring support (1 to 5). For medical evaluations, the clinical data suggestive of polypharmacy, sarcopenia, cognitive impairment, and frailty/oral frailty were obtained using questionnaires and physical examinations. Polypharmacy was defined as the use of six or more medications for > 1 month [8]. The contents of the medication were directly assessed by the medical staff using the medication handbook, not self-reported by the patients themselves. In particular, we examined patients who were prescribed with benzodiazepines and antihistamines and calculated the prescription rate. Sarcopenia was defined as age-related loss of muscle mass and strength [9]. Sarcopenia was evaluated using the finger-ring test [10] and 30-s chair stand test [11]. In the finger-ring test, the inability to encircle the calves indicated no risk of sarcopenia, the ability to encircle the calves indicated an increased risk of sarcopenia, and a gap observed between fingers and calves indicated a risk of sarcopenia. In the 30-s chair stand test, sarcopenia was diagnosed according to the age-specific index of standing numbers in 30 s. In this study, sarcopenia patients were defined as those who showed positive results on both finger-ring test and 30-s chair-stand test. Dementia, or cognitive dysfunction, is a chronic, progressive brain disease that results in higher brain dysfunction. In this study, the Mini-Cog test was used to diagnose this condition [12]. The term “frail” is derived from the word “frailty,” which means weakness, senility, or fragility, and is defined as a state of physical and mental deterioration due to aging [13]. The frailty screening index [14] and oral frailty [15] were used to screen for frailty. In addition, data on road traffic crashes history and the participants’ thoughts on driving licenses (future plans to renew or return licenses) were obtained.

### Statistical analysis

“Everyday drivers” were defined as those who answered they drive almost every day, while the rest were defined as “occasional drivers”; the background of these groups of drivers was compared. During the study period, data was prospectively collected without sample size calculation. Categorical variables were expressed as numbers, percentages, and odds ratios (ORs) with their 95% confidence intervals (CIs), which were assessed using the chi-square test or Fisher’s exact test, as appropriate. Continuous variables were expressed as median and interquartile range (IQR) and were analyzed using the Mann–Whitney test. The data were analyzed using EZR software, a graphic user interface for the R 3.5.2 software (The R Foundation for Statistical Computing, Vienna, Austria) [16]. A *P*-value of < 0.05 was considered significant.

### Ethics approval

Informed consent was obtained from all the participants. The study was approved by the Ethics Committee of Okayama University Graduate School of Medicine, Dentistry and Pharmaceutical Sciences and Okayama University Hospital’s Ethics Committee (approval no. 2002–031). This study was conducted according to the Declaration of Helsinki.

## Results

**Fig. 1:**
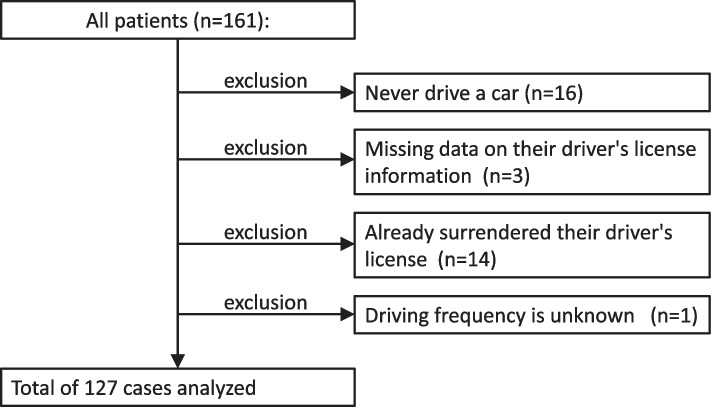
Selection of study patients

Data from 161 patients were obtained. Of them, (i) those who had never driven a car (16 patients), (ii) those who had missing data on their driver’s license information (three patients), (iii) those who had already surrendered their license (14 patients), and (iv) those whose driving frequency was unknown (one patient) were excluded. Thus, data from 127 patients were analyzed (Fig. 1). Raw data for the analysis is provided with supplementary file. The median (IQR) age was 73 (70–78) years; of the total participants, 82 were men (64.6%) and 45 were women (35.4%). With regard to the application status for nursing care insurance, 121 (95.3%) had not applied, 1 (0.8%) received insurance requiring help 1, and 3 (2.4%) received insurance requiring help 2. Among them, 77 patients (60.6%) drove almost every day, 38 (29.9%) drove once or a few times a week, 5 (3.9%) drove a few times a month, and 7 (5.5%) drove less than once a month. Consequently, the patients were classified into 77 everyday drivers and 50 occasional drivers (Table 1).

**Table 1:** Background data of the eligible patients

	Overall	Everyday driver	Occasional driver	*p* value
Total number	127	77	50	
Age (years), median [IQR]	73 [70, 78]	73 [70, 79]	74 [70, 76]	0.80
Sex, Male/Female (%)	82 (64.6) / 45 (35.4)	52 (67.5) / 25 (32.5)	30 (60.0) / 20 (40.0)	0.45
Status of nursing-care insurance level				
None	121 (95.3)	74 (96.1)	47 (94.0)	n.p
Requiring help 1	1 (0.8)	1 (1.3)	0	
Requiring help 2	3 (2.4)	0 (0)	3 (6.0)	
No data	2 (1.6)	2 (2.6)	0	

*n.p* Not performed

No difference was observed in the age, sex, and status of nursing-care insurance level between the two groups. With regard to the social factors, the frequency of going outside was significantly higher among everyday drivers. However, no differences were found in other factors such as the number of cohabiting family members, transportation most frequently used other than by car, walking time from home to the nearest station or bus stop, frequency of socializing with friends and peers, main source for daily news, daily concerns, eye doctor visits, orthopedic visits, and use of hearing aid (Supplementary Table 1).

The medical factors are summarized in Table 2. Overall, 27.6% of the patients were in polypharmacy, of whom 12.5 and 3.1% were taking benzodiazepine and anti-histamine drugs, respectively. The frequency of patients with polypharmacy was high among the everyday drivers, but no significant difference was observed (31.2% vs. 22.0%; OR: 0.63, 95% CI: 0.25–1.52]). With regard to sarcopenia, 17.3% of the patients who underwent the finger-wrist test and 50.4% of those who underwent the 30-s chair-stand test were found to be at risk for sarcopenia, with no significant difference between the two groups. Overall, the frequency of sarcopenia was higher among the everyday drivers (13.0% vs. 2.0%; OR: 0.14, 95% CI: 0.003–1.04). In the Mini-Cog test, 16.4% of the patients had cognitive impairment, but no difference was observed between the groups (18.2% vs. 14.0%; OR: 0.73, 95% CI: 0.23–2.14). Based on the frailty index, 63.0% of the patients were in the pre-frail state and 15.0% were in the frail state, without any statistical difference between the groups (15.6% vs. 14.0%; OR: 1.05, 95% CI: 0.26–4.1). Lastly, 54.3% of the patients were at high risk for oral frailty, but no difference was observed between the groups (50.6% vs. 60.0%; OR: 1.2, 95% CI: 0.41–3.58).

**Table 2:** Medical risk factors for road traffic crashes among the elderly drivers, by status of polypharmacy, sarcopenia, dementia, and frail

	Overall *N* = 127	Everyday driver *N* = 77	Occasional driver *N* = 50	OR (95% CI)	*p* value
**(1) Polypharmacy**					
Number of drugs prescribed more than 2 weeks, median [IQR]	4 [2, 6]	4 [2, 6]	3.5 [2, 5]	-	0.36
Polypharmacy^a^	35 (27.6)	24 (31.2)	11 (22.0)	0.63 (0.25–1.52)	0.35
-Including BZ drugs	16 (12.5)	11 (14.3)	5 (10.0)	0.67 (0.17–2.26)	0.59
-Including anti-histamine drugs	4 (3.1)	2 (2.6)	2 (4.0)	1.56 (0.11–22.2)	0.65
**(2) Sarcopenia**^**b**^					
No	114 (89.8)	66 (85.7)	48 (96.0)	*Reference*	
Yes	11 (8.7)	10 (13.0)	1 (2.0)	0.14 (0.003–1.04)	0.049
No data	2 (1.6)	1 (1.3)	1 (2.0)	-	
- **Finger-ring test**					
No risk of Sarcopenia	41 (32.3)	26 (33.8)	15 (30.0)	*Reference*	
Increased Risk of Sarcopenia	64 (50.4)	36 (46.8)	28 (56.0)	-	
Risk of Sarcopenia	22 (17.3)	15 (19.5)	7 (14.0)	0.81 (0.23–2.73)	0.79
- **30-s chair-stand test**					
No risk of Sarcopenia	61 (48)	33 (42.9)	28 (56.0)	*Reference*	
Risk of Sarcopenia	64 (50.4)	43 (55.8)	21 (42.0)	0.58 (0.26–1.26)	0.15
No data	2 (1.6)	1 (1.3)	1 (2.0)	-	
**(3) Dementia**					
- **Mini-cog**					
Not indicative of dementia (Score at 4 or 5)	106 (82.8)	63 (81.2%)	43 (86.0%)	*Reference*	
Risk of Dementia (Score at 0–3)	21 (16.4)	14 (18.2%)	7 (14.0%)	0.73 (0.23–2.14)	0.63
**(4) Frail**					
- **Frail Index**					
Not Frail (Score at 0)	28 (22.0)	18 (23.4)	10 (20.0)	*Reference*	
Pre-Frail (Score at 1–2)	80 (63.0)	47 (61.0)	33 (66.0)	-	
Frail (Score at ≥ 3)	19 (15.0)	12 (15.6)	7 (14.0)	1.05 (0.26–4.1)	1
- **Oral Frail**					
Low risk (Score at 0–2)	23 (18.1)	14 (18.2)	9 (18.0)	*Reference*	
Intermediate risk (Score at 3)	35 (27.6)	24 (31.2)	11 (22.0)	-	
High risk (Score at ≥ 4)	69 (54.3)	39 (50.6)	30 (60.0)	1.2 (0.41–3.58)	0.81

Percentages are given in the parentheses

*OR* Odds ratio, *CI* Confidential interval

^a ^Polypharmacy was defined as when patients prescribed 6 or more of drugs

^b ^Those fulfilling both of the finger-ring test and 30-s chair-stand test

Overall, 79 patients (62.2%) reported experiencing road traffic crashes in the past (Table 3). When the groups were compared, the frequency of road traffic crashes was higher among the occasional drivers than the everyday drivers, although no significant difference was observed (29.9% vs. 46.0%; OR: 2.1, 95% CI: 0.9–4.6). The intention to return the car license was significantly higher among the occasional drivers (2.6% vs. 14.0%; OR: 6.7 95% CI: 1.2–70.6, *p* = 0.024).

**Table 3:** History of road traffic crashes and their intention to return their car licence

	Overall *N* = 127	Everyday driver *N* = 77	Occasional driver *N* = 50	OR (95% CI)	*p* value
**Experience of accidents while driving?**					
Yes	79 (62.2)	23 (29.9)	23 (46.0)	2.1 (0.9–4.6)	0.09
No	46 (36.2)	53 (68.8)	26 (48.0)	*reference*	
No answer	2 (1.6)	1 (1.3)	1 (2.0)	-	
**Intention to return the car licence**					
Not intended	86 (67.7)	57 (74.0)	29 (58.0)	reference	
Considering	28 (22.1)	16 (20.8)	12 (24.0)	1.5 (0.6–3.8)	0.50
Planned to return it back	9 (7.1)	2 (2.6)	7 (14.0)	6.7 (1.2–70.6)	0.024
No answer	4 (3.2)	2 (2.6)	2 (4.0)	-	

Percentages are given in the parentheses

*OR* Odds ratio, *CI* Confidential interval

## Discussion

We investigated the prevalence of social and medical factors that can be associated with road traffic crashes among older patients aged ≥ 65 years. Notably, although less in number, 4 (3.1%) out of 127 participants were in a state of requiring level 1 or 2 assistance. By determining the current situation of older car drivers through this type of study, further measures can be improved at both national and medical field levels.

Previous literature has suggested that advanced age itself is a risk factor for road traffic crashes [17]. In addition, a wide variety of mental and physical dysfunctions inevitably observed during aging has been proven to yield a high incidence of road traffic crashes [6]. More specifically, dysfunctions in the sensory system (visual and auditory), central nervous system (stroke, depression, dementia, Parkinson’s disease, and insomnia), cardiovascular system (arterial hypertension, arrhythmia, coronary heart disease, and heart failure), musculoskeletal system (osteoarthritis and rheumatoid arthritis), diabetes mellitus, and polypharmacy as a consequence of the above potentially threatening traffic safety. Some of these factors are treatable, but most are refractory in older adults.

A causal association between multiple medications and road traffic crashes in older people has already been demonstrated [18]. In particular, use of narcotic analgesics combined with muscle relaxants, antidepressants, and antianxiety agents are known to lead to road traffic crashes. Although trends in polypharmacy have recently been ameliorated in Japan [19], it remains a major problem among older adults. Dementia disturbs the integrated cognitive functions [20] and attention [21], which are vital for safe driving. However, dementia patients continue to drive in the community, without proper physical fitness and appropriate driving behavior [22]. Frailty also increases the risk of road traffic crashes [23]. According to a Japanese observational study, pre-frailty/frailty individuals were highly at risk of experiencing road traffic crashes in the past year compared with robust individuals (OR: 3.74, 95% CI: 1.75–7.96) [24]. However, a recent review article did not show the clear relationship between road traffic crashes and sarcopenia [6]. The prevalence of medical factors in the present study was as follows: polypharmacy, 27.6%; dementia, 16.4%; and frail, 15.0%, indicating that older adults with medical factors are driving on a daily basis.

No differences were observed in the medical factors between the everyday drivers and occasional drivers, except for the high frequency of sarcopenia among everyday drivers, which was contrary to our expectations. However, occasional drivers had a higher rate of road traffic crashes history (46%), while 58% of them were not intended to return their licenses, suggesting that occasional drivers may have a potential risk for road traffic crashes in community.

A social opinion may be flipping in the restriction of car driving among older adults; however, the negative consequences should be considered. Recent cohort studies have suggested the relationship between driving cessation and an increased risk of functional deterioration in terms of physical, social, and mental health [25, 26]. With a limitation in out-of-home activity due to driving cessation, the alternative means of transportation are necessary for them to maintain their independence [27]. In addition to social aging, the number of older people living independently in rural areas continues to increase. Thus, this would be particularly true for those living in rural areas, since car driving is indispensable in the absence of an adequate public transportation network. Therefore, the advantages of car driving among aged individuals should also be considered.

The present study has a strength, in that the data were collected through physical examinations performed by physicians; thus, the results are considered credible. However, this study has several limitations. First, generalizability of the study results is difficult because of the small sample size. Second, considering the feasibility of the study, the finger-ring test and 30-s chair stand test were used to screen for sarcopenia, the Mini-Cog test for dementia, and the frailty screening index for frailty. However, the results of these screening tests should have been validated using more accurate scoring systems. Third, the data on the history of road traffic crashes lacked detailed information, especially the time of occurrence and situation. Therefore, we cannot clearly describe its relationship with medical factors. Forth, the generalizability needs to be carefully assessed, since the study included older patients visiting the hospitals. Despite these limitations, our attempt to identify the prevalence of medical factors among older drivers would be meaningful, as the results can objectively raise the potential issue in this aging society.

In summary, older people with medical factors drive cars on a daily basis in our community. To avoid road traffic crashes caused by older drivers, further strict screenings and evaluations may be required. At the same time, protecting the lives of older adults living in rural areas should be taken into account. Due to the limitations mentioned above, a future longitudinal study with a larger sample size and a comparison to non-drivers, including an investigation on health damage by not driving, is required.

## Supplementary Information

**Additional file 1. **Raw data for the analysis.

**Additional file 2: Supplementary Table 1.** Potential social risk factors for road traffic crashes by car driving among the older people.

## Data Availability

All data generated or analysed during this study are included in this published article [and its supplementary information files].
