# Supplementary material for: Prevalence of medical factors related to aging among older car drivers: a multicenter, cross-sectional, descriptive study
Source: BMC Geriatr. 2022 Oct 11;22:792. doi: 10.1186/s12877-022-03490-w (PMC9555182; doi:10.1186/s12877-022-03490-w)
Supplement: Supplementary file 2 — Additional file 2: Supplementary Table 1. Potential social risk factors for road traffic crashes by car driving among the older people. [file 12877_2022_3490_MOESM2_ESM.docx]

**Supplementary Table 1. Potential social risk factors for road traffic crashes by car driving among the older people**

|  | Overall | Everyday driver | Occasional driver | *p* value |
| --- | --- | --- | --- | --- |
| Frequency of going outside | | | | |
| Less than 2〜3 times per week  Every day  No data | 46 (36.2)  76 (59.8)  5 (3.9) | 14 (18.2)  59 (76.6)  4 (5.2) | 33 (66.0)  16 (32.0)  1 (2.0) | <0.001 |
| Number of cohabiting family members | | | | |
| None  1  2  3  4  5  6  No data | 20 (15.7)  64 (50.4)  23 (18.1)  8 (6.3)  3 (2.4)  6 (4.7)  2 (1.6)  1 (0.8) | 13 (16.9)  39 (50.6)  12 (15.6)  6 (7.8)  2 (2.6)  2 (2.6)  2 (2.6)  1 (1.3) | 7 (14.0)  25 (50.0)  11 (22.0)  2 (4.0)  1 (2.0)  4 (8.0)  0  0 | n.p. |
| Transportation most frequently used other than by car | | | | |
| Walk  Bicycle  Motorcycle  Train / Bus  No data | 91 (71.7)  19 (15.0)  8 (6.3)  4 (3.1)  5 (3.9) | 57 (74.0)  12 (15.6)  3 (3.9)  2 (2.6)  3 (3.9) | 34 (68.0)  7 (14.0)  5 (10.0)  2 (4.0)  2 (4.0) | 0.69 |
| Walking time from your home to the nearest station or bus stop | | | | |
| 5 minutes  10 minutes  30 minutes  No data | 38 (29.9)  44 (34.6)  38 (29.9)  7 (5.5) | 21 (27.3)  23 (29.9)  29 (37.7)  4 (5.2) | 17 (34.0)  21 (42.0)  9 (18.0)  3 (6.0) | 0.06 |
| Do you have opportunities to communicate with friends, such as senior citizens' association or club activities? | | | | |
| No  Yes  No data | 58 (45.7)  67 (52.8)  2 (1.6) | 34 (44.2)  41 (53.2)  2 (2.6) | 24 (48.0)  26 (52.0)  0 | 0.86 |
| How do you find out the news of the day? | | | | |
| TV  Radio  News paper  Internet | 114 (89.8)  12 (9.4)  68 (53.5)  17 (13.4) | 70 (90.9)  8 (10.4)  40 (51.9)  10 (13.0) | 44 (88.0)  4 (8.0)  27 (54.0)  7 (14.0) | 0.97 |
| Concern you worry about the most | | | | |
| My own health  Family  Financial affair  Human relations  Nothing special  No data | 70 (55.1)  15 (11.8)  6 (4.7)  1 (0.8)  31 (24.4)  4 (3.1) | 41 (53.2)  8 (10.4)  3 (3.9)  1 (1.3)  21 (27.3)  3 (3.9) | 29 (58.0)  7 (14.0)  3 (6.0)  0  10 (20.0)  1 (2.0) | 0.80 |
| Do you visit an eye doctor? | | | | |
| No  Yes  No data | 87 (68.5)  39 (30.7)  1 (0.8) | 57 (74.0)  19 (24.7)  1 (1.3) | 30 (60.0)  20 (40.0)  0 | 0.08 |
| Do you visit an orthopedic clinic? | | | | |
| No  Yes | 98 (77.2)  29 (22.8) | 57 (74.0)  20 (26.0) | 41 (82.0)  9 (18.0) | 0.39 |
| Do you use a hearing aid? | | | | |
| No  Yes | 117 (92.1)  10 (7.9) | 70 (90.9)  7 (9.1) | 47 (94.0)  3 (6.0) | 0.74 |

n.p., not performed. Chi-square test and Fisher’s test were used as appropriate. Percentages are given in the parentheses.
